# Prolonged visual perceptual changes induced by short-term dyadic training: The roles of confidence and autistic traits in social learning

**DOI:** 10.1016/j.isci.2024.111716

**Published:** 2024-12-30

**Authors:** Bin Zhan, Yujie Chen, Rui Wang, Yi Jiang

**Affiliations:** 1 State Key Laboratory of Cognitive Science and Mental Health, Institute of Psychology, Chinese Academy of Sciences, Beijing 100101, China; 2 Department of Psychology, University of Chinese Academy of Sciences, Beijing 100049, China

**Keywords:** neuroscience, behavioral neuroscience, sensory neuroscience

## Abstract

As social creatures, we are naturally swayed by the opinions of others, which largely shape our attitudes and preferences. However, whether social influence can directly impact our visual perceptual experience remains debated. We designed a two-phase dyadic training paradigm where participants first made a visual categorization judgment and then were informed of an alleged social partner’s choice on the same stimulus. Results demonstrated that social influence significantly modified participants’ subsequent visual categorizations, even when they had been well-trained prior to the dyadic training. This effect persisted for an extended period of up to six weeks. Diffusion model analysis revealed that this effect stemmed from perceptual processing more than mere response bias, and its strength was inversely related to the participants’ confidence and autistic-like tendencies. These findings offer compelling evidence that our perceptual experiences are deeply influenced by social factors, with individual confidence and personality traits playing significant roles.

## Highlights

• Observing others’ choices can lead to persistent changes in visual perception
• Confidence influences how social information is integrated into perceptual decisions
• Individuals with low autistic tendencies are more susceptible to social influence
• Social context involving real-time human interactions exerts greater social influence

## Graphical abstract

**Figure:**
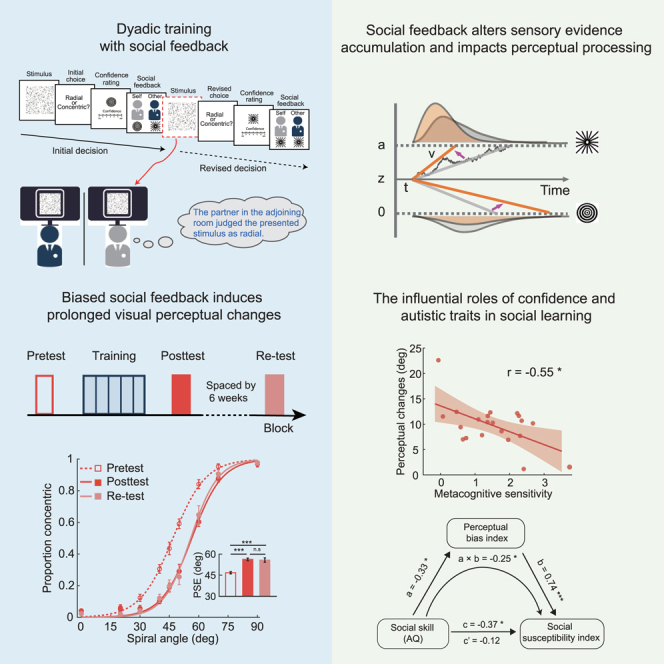

## Introduction

People often consider themselves independent thinkers, free to make their own choices. However, our minds and actions are inevitably influenced by observing others’ opinions or behaviors, either explicitly or implicitly, a phenomenon known as social influence.^1^^,^^2^ For example, our preferences for goods are influenced by what other people prefer,^3^ and observing peers’ choices can alter our risk preferences.^4^ Apart from these deliberate decisions that rely on high-level cognitive functions,^5^ social influence also extends to low-level perceptual decision-making.^6^^,^^7^ Recent work has shown that sharing information between group members significantly modifies the collective decisions on simple visual perceptual tasks.^8^^,^^9^

Perceptual decision-making is typically regarded as a process that largely mirrors the physical reality of the external world. The brain extracts sensory signals represented in the sensory cortex and transforms these signals across sensorimotor and motor areas to construct veridical percepts and guide adaptive behaviors.^10^ Perceptual processing is usually thought to be relatively automatic and stable.^11^ Nonetheless, it has been reported that individuals within a group tend to conform to the majority opinion in perceptual tasks, even though the majority are blatantly wrong.^12^^,^^13^ Whether social influence can directly impact our perceptual processing remains debated. Several studies have demonstrated that social influence can dynamically alter the way people perceive incoming sensory information.^14^^,^^15^^,^^16^ However, some other work has shown that social influence modifies decision process through shifting subjective response tendencies in line with social norms but not affecting perceptual experience.^17^ That is, people may change their reported percepts or responses merely for social acceptance while still internally adhering to their initial percepts, suggesting that social influence might only lead to a transient response change rather than an enduring perceptual alteration. Here, we aim to investigate whether social influence engenders a genuine perceptual change that can be long-lasting and persistent, even when social context is absent. To the best of our knowledge, there has been no investigation of social influence on perceptual decisions over a long period.

**Figure 1:**
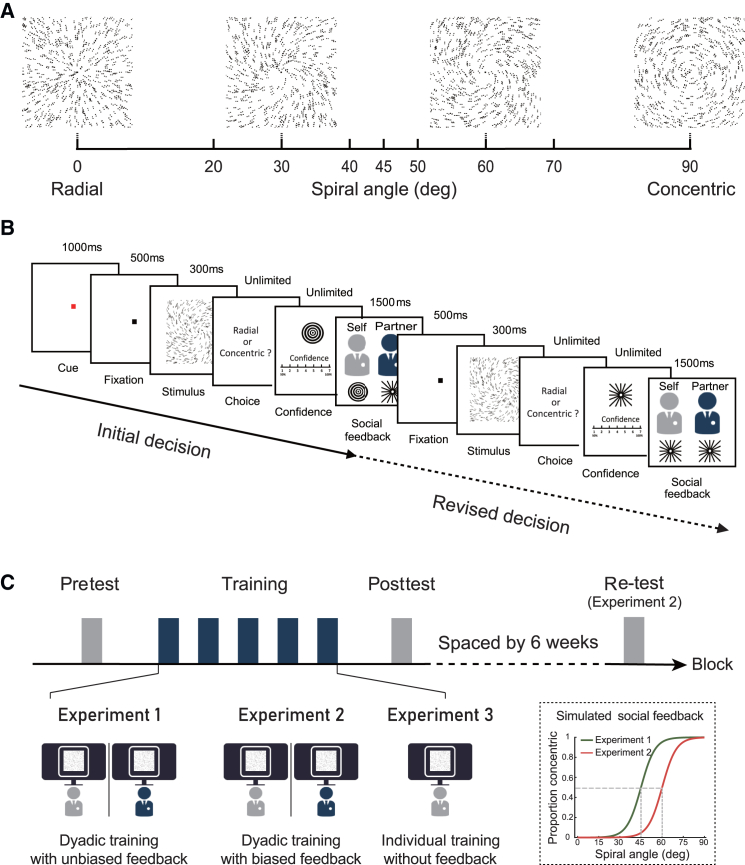
Stimuli and experimental design (A) Stimuli: four example Glass pattern stimuli (100% signal) at spiral angles of 0°, 30°, 60°, and 90°. The testing spiral angles are indicated by black bars. (B) Trial design in dyadic training. Each trial started with a red cue and comprised two decision phases. Observers were first presented with a Glass pattern stimulus and were asked to make a categorical judgment (radial vs. concentric) as well as rate their confidence in that decision on a discrete scale from 1 to 7 (initial decision phase). They were informed of their partner’s choices displayed underneath their corresponding portraits. Then, observers viewed a second Glass pattern stimulus at the same spiral angle as in the initial decision phase and reported their second judgment and confidence (revised decision phase). (C) Experimental protocol and design in experiments 1–3. All the observers underwent three stages (pretest, training, and posttest) over multiple task blocks on the same day. A subgroup of participants in experiment 2 had an additional re-test six weeks after training. In experiments 1 and 2, observers were paired to perform the visual categorization task simultaneously seated in two adjacent rooms and each one was informed of an alleged social partner’s choice on the same stimulus during training. The peers’ responses were simulated using an algorithm based on an unbiased (45°, experiment 1) or shifted (60°, experiment 2) categorization boundary. Observers in experiment 3 were trained individually without any feedback. During the test phases, observers performed the same visual categorization task but no feedback was provided.

In the present study, we adopted a dyadic training paradigm in which participants were trained on a visual categorization task in pairs, and each of them was informed of an alleged social partner’s choice on the same stimuli (i.e., social feedback) during training. We tested individuals’ ability to categorize the visual patterns before and immediately after the social learning, with a re-test six weeks later (Figure 1), which allowed us to examine the stability of perceptual changes after training with social feedback. In addition, we modeled observers’ behavioral responses during the dyadic training phase using a drift-diffusion model (DDM)^18^ to characterize the cognitive processes involved in visual categorization and evaluate the social influence on these processes. In the framework of DDM, perceptual decision-making is described as a process that brain accumulates noisy sensory evidence over time until a certain amount of information (i.e., decision threshold) is obtained to reach a decision.^19^ Specifically, this model offers a useful tool to disentangle whether social influence is accompanied with a modulation of perceptual processing (i.e., the uptake of sensory evidence is affected), a bias at judgmental level (i.e., priori response preference is shifted), or both.^15^^,^^17^ We reason that long-lasting perceptual changes exerted by observing others’ choices might reflect the internalization of social influence through modulating sensory processes, whereas an outward sign of public compliance with others’ views is more likely to be transient response changes and easily fade away when social context is absent.

Furthermore, we seek to identify the critical factors that modulate perceptual decisions under social influence. Decisions are often accompanied with a certain degree of confidence which reflects an internal estimation of the information gathered from the environment.^20^ Recent work has suggested that confidence, as a metacognitive process of uncertainty monitoring, plays an important role in collecting evidence for a choice, guiding subsequent actions, and exploring alternatives.^21^^,^^22^ On the other hand, individuals usually exhibit consistent, context-independent differences in their propensities to utilize social information.^23^ Patient studies have reported that children and adults with autism spectrum disorder conform less to others than the neurotypical group.^24^^,^^25^ Hence, we reason that the effect of social influence might relate to an internal metacognitive process (i.e., confidence) and individual traits (e.g., autistic-like tendency). In this study, we measured participants’ choices and their confidence before and after receiving social feedback for each trial during training. This two-phase design allowed us to dynamically track participant responses over time and characterize how personal and social information was integrated into subsequent perceptual decisions. We further assessed individual differences in their susceptibility to social information when making decisions under uncertainty, and explored whether it relates to personality traits.

## Results

### Social feedback modulates perceptual decision-making

Three groups of observers (experiment 1–3) were trained to make categorical judgments on visual stimuli that morphed between radial and concentric patterns (Figure 1A). To investigate whether and how social contexts affect perceptual decision-making, we designed a dyadic training paradigm in which pairs of observers were recruited to perform this visual categorization task simultaneously and each one was informed of their partner’s responses during training (Figure 1B). Unbeknownst to the participants, the peers’ responses were simulated using an algorithm based on an unbiased (45° spiral angle, experiment 1) or shifted (60° spiral angle, experiment 2) categorization boundary, referred to as unbiased and biased social feedback, respectively (Figure 1C). This paradigm allowed us to conduct training on one arbitrary category boundary and then test experience-dependent changes in perceptual sensitivity and categorization boundary. An additional individual training group in which observers were trained alone without any social interactions was included as a baseline experiment (experiment 3). All observers completed five training blocks (450 trials) which lasted about 1.5 h. No error feedback was provided in these experiments.

**Figure 2:**
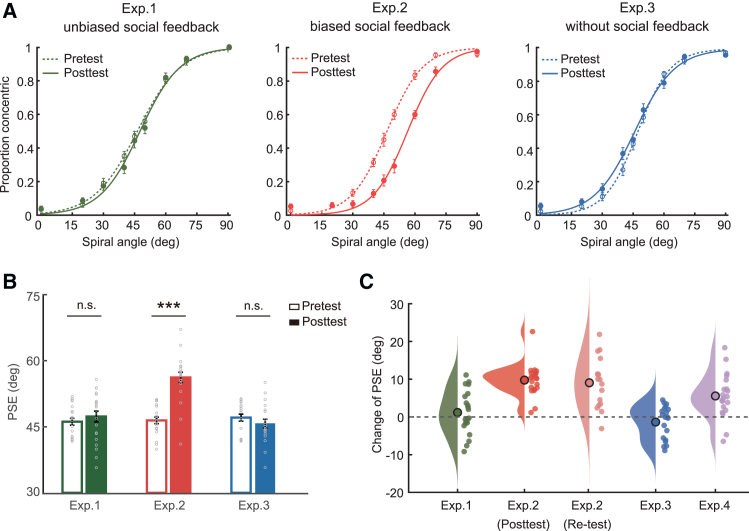
Social feedback shapes visual categorization (A) The proportion of responses in which observers indicated the Glass pattern as concentric is plotted as a function of spiral angle from experiment 1–3. Data are shown for the pretest (dash lines) and the posttest (solid lines). (B) Visual categorization boundaries indicated by the subjective equality points (PSE) before and after training. Each color corresponds to a unique experiment. Individual data are plotted as gray circles. (C) The shift of categorization boundary (i.e., change of PSE) is shown separately across experiment 1 to experiment 4. The small colored circles and large open circles respectively represent individual data and the means of the perceptual changes. The distribution of individual data (shaded region) was plotted for each experiment using the Raincloud plots package.^26^ Error bars show ±1 SEM. ∗∗∗*p* < 0.001, n.s.: nonsignificant.

We tested observers’ ability to categorize global form patterns as radial or concentric before and after training and plotted their performance as a function of stimulus spiral angle (Figure 2A). Our results showed that biased social feedback significantly shifted the observers’ criteria for categorical judgments. A repeated-measures ANOVA comparing categorization boundaries (50% point on the psychometric function, that is the point of subjective equality, PSE) across tests (pre- and post-training tests) and groups (experiments 1, 2, and 3) showed a significant interaction of test and experimental group (*F*(2,59) = 30.19, *p* < 0.001, *η*^*2*^_*p*_ = 0.51). Before training, observers’ categorization boundaries were not significantly different between experimental groups (one-way ANOVA, *F*(2,59) = 0.38, *p* = 0.687, *η*^*2*^_*p*_ = 0.01) and matched closely the mean of the physical stimulus space (45° spiral angle). After training, biased social feedback has significantly shifted the observers’ criteria for categorization toward the 60° boundary (experiment 2: PSE at pretest vs. posttest: 46.45 vs. 56.20, 95% confidence interval [CI] for the mean difference = [7.82, 11.68], Cohen’s *d* = 2.30, *t*(20) = 10.53, *p* < 0.001), in contrast to no significant change on perceptual boundary observed for groups trained with unbiased social feedback or trained alone (experiment 1: PSE at pretest vs. posttest: 46.15 vs. 47.32, 95% CI for the mean difference = [-1.42, 3.78], Cohen’s *d* = 0.21, *t*(20) = 0.94, *p* = 0.356; experiment 3: pretest vs. posttest: 47.08 vs. 45.72, 95% CI for the mean difference = [-3.40, 0.67], Cohen’s *d* = −0.31, *t*(19) = −1.40, *p* = 0.177, Figure 2B). We also observed a gradual shift of perceptual boundary over training blocks (Figure S1). However, comparing the perceptual sensitivities (i.e., the slopes of psychometric functions) before and after training across groups showed neither significant main effects (Test: *F*(1,59) = 0.02, *p* = 0.883, *η*^*2*^_*p*_ = 3.68 × 10^−4^; Group: *F*(2,59) = 2.57, *p* = 0.085, *η*^*2*^_*p*_ = 0.08) nor the interaction (*F*(2,59) = 1.13, *p* = 0.331, *η*^*2*^_*p*_ = 0.04).

To further examine whether the shift in the observers’ categorization boundary could be maintained over time, seventeen participants of experiment 2 were called back for an additional test six weeks after training. Surprisingly, the change of PSE was retained and did not significantly differ from that tested immediately after training (mean PSEs at pretest, posttest, and re-test were 46.73, 56.24, and 55.78 respectively, ANOVA, *F*(2,32) = 31.46, *p* < 0.001, *η*^*2*^_*p*_ = 0.66, paired-samples t test, 95% CI for the difference of means between posttest and re-test = [-3.79, 2.87], Cohen’s *d* = −0.07, *t*(16) = −0.29, *p* = 0.773, Figure 2C), suggesting social influence on perceptual decisions was sustained for a prolonged time.

Considering the categorization criteria in our paradigm may be ambiguous to naive participants that they were easily altered by social feedback, we ran an additional experiment to test whether experienced participants with clear categorization boundaries are similarly influenced by social feedback (experiment 4). Another group of observers was first trained individually to categorize global form patterns for multiple days (1,800–3,600 trials in total) and then underwent a dyadic training session with biased social feedback (30° or 60° spiral angle) following the same procedure as in experiment 2. Intensive individual training associated with trial-by-trial unbiased error feedback improved observers’ perceptual sensitivity in categorical judgments, which was confirmed by a significant increase in the slope of the psychometric function after training (after vs. before, 0.18 vs. 0.12, 95% CI for the mean difference = [0.03, 0.09], Cohen’s *d* = 1.01, *t*(19) = 4.50, *p* < 0.001), and the categorization boundaries (46.43 ± 2.43°) matched closely the mean of the physical stimulus space. Interestingly, mere 90-min dyadic training with biased social feedback resulted in a significant, though relatively weak, shift in observers’ categorization criteria toward the partners’ boundaries (mean difference = 5.55, 95% CI for the mean difference = [2.78, 8.33], Cohen’s *d* = 0.94, *t*(19) = 4.19, *p* < 0.001, Figure 2C), affirming an irresistible social influence even in well-trained individuals.

### Confidence and social feedback influence subsequent behavioral responses

**Figure 3:**
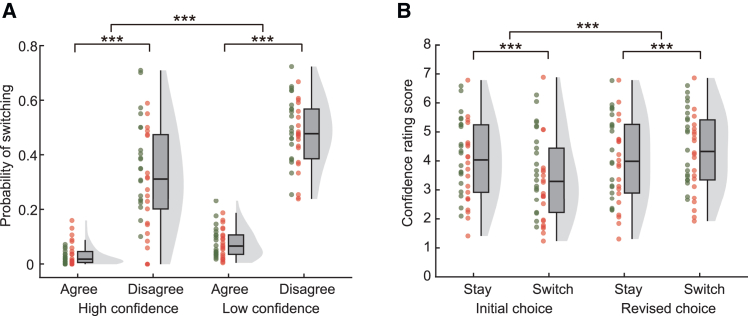
Behavioral results in the revised decisions and confidence during dyadic training (A) Boxplots illustrating the probability of choice switching during the revised decisions are presented as a function of social influence (agreement vs. disagreement) and initial confidence (high vs. low). (B) Confidence ratings for initial and revised choices are separated by different revised decisions (i.e., to stay or switch initial choices) when facing disagreement. Data are averaged across participants in experiment 1 and experiment 2 as no significant group difference is observed. Individual data are plotted and the color of the dots indicates the experimental group (green dots for experiment 1; red dots for experiment 2). Data present sample distributions, along with the quantiles and median scores for each condition. ∗∗∗*p* < 0.001.

Decisions that we are making are accompanied with some degree of confidence on whether the concurrent judgments are correct. Recent work has suggested that confidence, as an internal metacognitive process of uncertainty monitoring, is crucial in revising one’s decisions.^21^^,^^22^^,^^27^ In this study, we evaluated observers’ confidence in their perceptual choices over training. In each trial, observers made perceptual judgments twice for the same level of stimulus and each judgment was followed by a graded confidence rating (Figure 1B). This two-phase procedure allowed us to examine how individuals’ confidence associated with social feedback influenced their subsequent perceptual decision-making. We separated trials based on observers’ initial confidence (high vs. low confidence compared to the mean value) or social feedback (agreement vs. disagreement) and test whether individual choices were changed in the revised decision phase (Figure 3A). Our results demonstrated that observers were less likely to change their minds when holding higher confidence in their initial choices or when receiving inconsistent responses from partners. A 3-way ANOVA with confidence (high and low), social feedback (agreement and disagreement), and group (experiments 1 and 2) as factors on the probability of choice switching (*P*_*switch*_) showed significant main effects of confidence (*F*(1,40) = 58.47, *p* < 0.001, *η*^*2*^_*p*_ = 0.59) and social feedback (*F* (1,40) = 337.00, *p* < 0.001, *η*^*2*^_*p*_ = 0.89). More importantly, there was a significant interaction between initial confidence and social feedback (*F*(1,40) = 22.22, *p* < 0.001, *η*^*2*^_*p*_ = 0.36). Post-hoc analyses revealed that inconsistent responses from partners leaded to higher probability of choice switching and this effect was more pronounced when observers showed lower confidence in their initial choices (high confidence, disagreement vs. agreement, mean difference = 0.29, 95% CI for the mean difference = [0.23, 0.34], Cohen’s *d* = 1.63, *t*(41) = 10.56, *p* < 0.001; low confidence, disagreement vs. agreement, mean difference = 0.40, 95% CI for the mean difference = [0.36, 0.44], Cohen’s *d* = 3.27, *t*(41) = 21.22, *p* < 0.001), indicating that confidence plays a critical role in the use of social sources for perceptual decision-makings. The lack of a significant three-way interaction (*F*(1,40) = 2.37, *p* = 0.131, *η*^*2*^_*p*_ = 0.06) and the main effect of group (*F*(1,40) = 2.60, *p* = 0.115, *η*^*2*^_*p*_ = 0.06) suggested similar trends observed in both groups.

Comparing observers’ confidence rating scores between the initial and revised decision phases across experimental groups (experiments 1 and 2) revealed a significant boost in confidence through revision (*F*(1,40) = 40.09, *p* < 0.001, *η*^*2*^_*p*_ = 0.50). Notably, this boost in confidence was observed even in the face of disagreement (*F*(1,40) = 31.02, *p* < 0.001, *η*^*2*^_*p*_ = 0.44). In particular, we compared the self-rated confidence for different revised decisions (i.e., whether to follow the opinions of others and switch the choices). Interestingly, there was a significant interaction between revised choice (stay vs. switch) and decision phase (*F*(1,40) = 73.93, *p* < 0.001, *η*^*2*^_*p*_ = 0.65) in a three-way ANOVA (revised choice × decision phase × group) (Figure 3B). Post-hoc analyses revealed that when observers showed lower confidence in their initial decisions, they tended to take their partners’ advice and switch choices, whereas they adhered to their initial choices when holding higher confidence (initial confidence, stay vs. switch, mean difference = 0.58, 95% CI for the mean difference = [0.44, 0.72], Cohen’s *d* = 1.29, *t*(41) = 8.34, *p* < 0.001). Following partners’ opinions substantially boosted observers’ confidence, making them even more confident in these revisions compared to sticking to their original choices (revised confidence, stay vs. switch, mean difference = −0.26, 95% CI for the mean difference = [-0.40, −0.13], Cohen’s *d* = −0.63, *t*(41) = −4.08, *p* < 0.001). Taken together, our results demonstrate that individuals are more susceptible to social influence when lacking confidence, and conforming to the opinions of others significantly boosts their confidence in perceptual decisions.

### Drift-diffusion modeling reveals social influence leads to changes in perceptual processing

**Figure 4:**
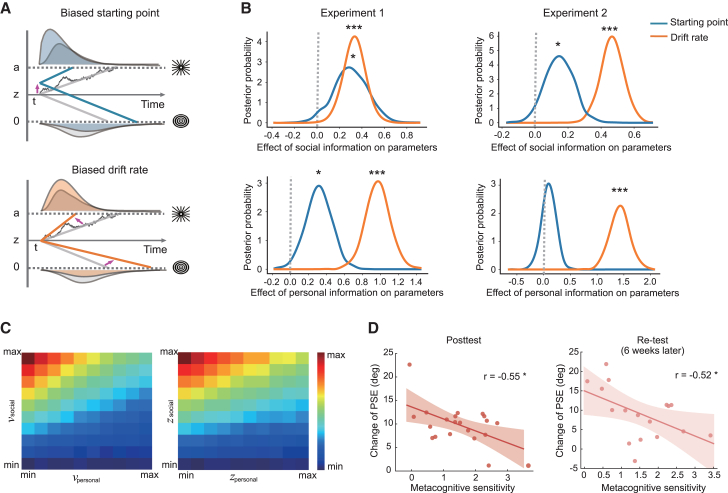
Drift diffusion modeling results (A) Illustration of how personal or/and social information may modulate perceptual decision-making within the DDM framework. The DDM can capture decision bias through a shift in the starting point (*z*, upper panel) and/or through an alteration in the drift rate (*v*, lower panel) of evidence accumulation toward the different decision thresholds (*a*). (B) Posterior distributions of model parameters (starting point and drift rate) for the best-fitting model. The dependencies of the starting point (blue lines) and the drift rate (orange lines) on social information (upper panel), and personal information (lower panel) in experiment 1 (lefthand) and experiment 2 (righthand) are presented. More than 95% of the distribution was greater than 0, indicating significant positive effect on these parameters. (C) Visualization of change of PSE from model simulations, values as a function of various dependencies of the drift rate (*v*, lefthand panel) and the starting point (*z*, righthand panel) on personal (initial confidence, x axis) or social information (y axis). Hotter colors indicate greater perceptual changes. (D) Negative correlations between perceptual change (i.e., change of PSE) and metacognitive sensitivity at both posttest and re-test (six weeks later). ∗*p* < 0.05, ∗∗∗*p* < 0.001.

When making a decision in the cluttered world imbued with uncertainty, individuals may not only count on the personal information gathered from sampling the physical environment but also utilize the social information provided by others to improve decision accuracy. To further explore the underlying cognitive process through which personal information (i.e., observers’ confidence level in their initial choices) and social information (i.e., partner’s choices) are integrated into subsequent perceptual decision-making, we applied a DDM analysis to behavioral responses during dyadic training in experiments 1 and 2. Note that only the revised decisions were being modeled, as the observers’ choices and confidence ratings from the initial decision stage before receiving any social feedback are considered representative of individuals’ subjective independent judgments.^16^^,^^22^ According to the DDM, perceptual decision-making between two alternatives can be described as a dynamic process in which sensory evidence is accumulated over time until a decision threshold is reached.^18^^,^^28^ In our study, we reasoned that individuals may incorporate personal and social information by promoting a biased evidence accumulation process toward the boundary associated with their initial option or the option favored by their partners. In particular, we considered two potential mechanisms accounting for this biased process, that is, personal and/or social information may shift the priori response preference so that less supportive evidence is needed (i.e., a response bias) or affect the uptake of sensory evidence (i.e., perceptual bias). Critically, DDM can be used to disentangle these two processes by decomposing behavioral data (i.e., choices and RT distributions) into two key parameters: starting point (*z*) for a prior response bias, and drift rate (*v*) for speed of evidence accumulation (Figure 4A).

To this end, we tested 10 candidate models that embodied these different predictions and were composed of various combinations of the two key model parameters varying with personal and social information (see details in STAR Methods). In the baseline model (model 1), neither the starting point nor the drift rate was affected by personal and social information, but the drift rate was allowed to vary with the stimulus spiral angle. The remaining models differed to examine whether the starting point or/and drift rate were affected by personal information (models 2–4), social information (models 5–7), or both (models 8–10). Model fits were assessed using the deviance information criterion (DIC).^29^^,^^30^ The best-fitting model is the full model (model 10, as indicated by the lowest DIC score) in experiments 1 and 2, incorporating dependencies of the starting point and the drift rate on both personal and social information. We took several steps to validate this best-fitting model (see details in model analysis, Figures S2–S6). These model simulation analyses affirmed that the full model well captured the key cognitive processes involved in perceptual decision-making under social influence.

We extracted the posterior distributions of the starting point and the drift rate estimated by the full model to quantify the extent to which personal and social information affected these two parameters (Figure 4B). Our results demonstrated that personal information had significant positive effects on the drift rate (experiment 1: *p*(*v*_personal_ > 0) > 0.999, mean = 0.96, 95% credible interval = 0.69–1.22; experiment 2: *p*(*v*_personal_ > 0) > 0.999, mean = 1.36, 95% credible interval = 1.02–1.72) and the starting point (experiment 1: *p*(*z*_personal_ > 0) = 0.990, mean = 0.33, 95% credible interval = 0.05–0.61; experiment 2 showed a similar but non-significant positive tendency: *p*(*z*_personal_ > 0) = 0.792, mean = 0.10, 95% credible interval = −0.16 – 0.35), suggesting that individuals incorporated their personal information via starting closer to the threshold of initially chosen option, and higher confidence resulted in more pronounced biased sensory evidence accumulation. Similarly, there were significant positive effects of social information on the drift rate (experiment 1: *p*(*v*_social_ > 0) > 0.999, mean = 0.33, 95% credible interval = 0.14–0.52; experiment 2: *p*(*v*_social_ > 0) > 0.999, mean = 0.45, 95% credible interval = 0.31–0.59) and the starting point (experiment 1: *p*(*z*_social_ > 0) = 0.975, mean = 0.30, 95% credible interval = −4.65 × 10^−4^ – 0.59; experiment 2: *p*(*z*_social_ > 0) = 0.959, mean = 0.14, 95% credible interval = −0.02 – 0.31). Taken together, personal and social information influenced subsequent perceptual decision-making in two ways: adjusting the initial response criteria and inducing selective accumulation of sensory evidence. A comparison of the estimated parameters showed that the effect of either personal or social information on the drift rate was more pronounced than that on the starting point (experiment 1: *p*(*v*_personal_ > *z*_personal_) = 0.999, *p*(*v*_social_ > *z*_social_) = 0.568; experiment 2: *p*(*v*_personal_ > *z*_personal_) > 0.999, *p*(*v*_social_ > *z*_social_) = 0.996), suggesting that the perceptual bias played a more important role in integrating personal and social information into perceptual decisions.

Moreover, the DDM allowed us to specify how individuals’ categorization boundaries were shaped by personal and social information. We implemented the simulation by systematically varying the four key parameters, *v*_personal_, *v*_social_, *z*_personal_, and *z*_social_, which represented the influences exerted by personal and social information on perceptual processing and response preference, respectively. As shown in Figure 4C, the perceptual change was larger when *v*_social_ or *z*_social_ was higher, and *v*_personal_ or *z*_personal_ was lower. Consistent with our empirical data, the simulation results implied that if observers felt less confident in their decisions, a selective evidence accumulation process in favor of others’ choices was promoted, resulting in a greater perceptual change induced by social influence. Hence, we speculated that such confidence judgments, which reflect the ability to recognize one’s own successful choices, may predict the perceptual change exerted by social influence. Specifically, we calculated the extent to which confidence discriminates between correct and incorrect responses, namely metacognitive sensitivity,^31^ avoiding the overall level of confidence expressed bias. As expected, in experiment 2, we found significant negative correlations between metacognitive sensitivity and changes of PSEs which were tested immediately after training (*r*(19) = −0.55, *p* = 0.010) and even six weeks later (*r*(15) = −0.52, *p* = 0.033) (Figure 4D). These results highlight that confidence is a robust predictor of perceptual changes caused by social influence, implying that the metacognitive process guides individuals to selectively assimilate the opinions of others into perceptual decision-making.

### Individual differences in the integration of personal and social information

**Figure 5:**
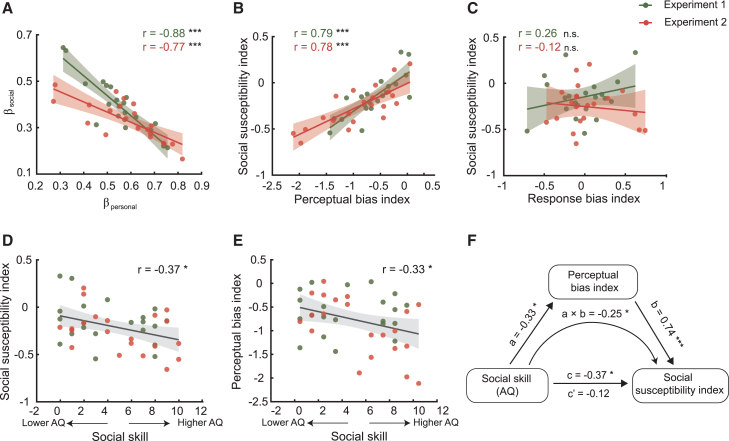
Individual differences in integrating personal and social information and its correlations with autistic traits (A) The influences of personal and social information on revised confidence, indicated by β_personal_ and β_social_ respectively, are negatively correlated. (B and C) Correlating individual susceptibility to social influence with perceptual bias and response bias. The social susceptibility index is associated with perceptual bias index but not response bias index. (D and E) Correlating autistic traits with individual susceptibility to social influence and perceptual bias index. The social skill factor of the AQ is negatively correlated with social susceptibility index and perceptual bias index. A lower AQ score in the social skill domain indicates better social proficiency. (F) Results of mediation analysis. The mediation model included AQ (social skill domain of the AQ) as the independent variable, social susceptibility index as the dependent variable, and perceptual bias index as the mediator. The influence of autistic tendency on social susceptibility was fully mediated by its perceptual processing. Social susceptibility index: β_social_˗β_personal_; Perceptual bias index: *v*_social_˗*v*_personal_; response bias index: *z*_social_˗*z*_personal_. Data are plotted across participants in experiment 1 and experiment 2, and the color of dots indicates the experimental group. ∗*p* < 0.05, ∗∗∗*p* < 0.001, n.s.: nonsignificant.

We next investigated the individual differences in their susceptibility to social influence. As the final responses expressed by revised choices and confidence (i.e., belief updating) have merged the personal information gathered during initial decision phase and the social information gathered by observing partners’ choices, we performed a multiple linear regression analysis to quantify the contributions of these two information sources at individual level. We extracted two regression coefficients, β_personal_ and β_social_, reflecting the extent to which individuals were influenced by personal information and social information, respectively. Results showed that both personal (experiment 1, *t*(20) = 21.45, *p* < 0.001; experiment 2, *t*(20) = 18.06, *p* < 0.001) and social information (experiment 1, *t*(20) = 15.92, *p* < 0.001; experiment 2, *t*(20) = 17.82, *p* < 0.001) sources contributed to revised confidence. Interestingly, the two coefficients were negatively correlated (experiment 1, Pearson’s *r*(19) = −0.88, *p* < 0.001; experiment 2, Pearson’s *r*(19) = −0.77, *p* < 0.001) (Figure 5A), which is in line with our previous findings that individuals are more likely to be influenced by others when they are uncertain about their own choices. Next, we computed a social susceptibility index (SSI) that indicated individual susceptibility to social influence versus personal information by calculating the difference between the two coefficients (β_social_˗β_personal_) due to the observed antagonistic contribution of personal and social factors to belief updating. We then asked whether this individual susceptibility to social influence is accounted for by the response preference or perceptual processing. We extracted the individual parameters estimated from the DDM model and derived a perceptual bias index (*v*_social_˗*v*_personal_) and a response bias index (*z*_social_˗*z*_personal_). We found that SSI was highly correlated with perceptual bias index (Figure 5B, experiment 1, Pearson’s *r*(19) = 0.79, *p* < 0.001; experiment 2, Pearson’s *r*(19) = 0.78, *p* < 0.001) but not with response bias index (Figure 5C, experiment 1, Pearson’s *r*(19) = 0.26, *p* = 0.251; experiment 2, Pearson’s *r*(19) = −0.12, *p* = 0.597), indicating that integration of personal and social information in belief updating essentially stemmed from sensory evidence accumulation process rather than response preference.

Finally, we explored whether individual susceptibility to social influence relates to personality traits. We measured observers’ autistic traits using an adapted version of the autism-spectrum quotient (AQ).^32^ Combining data across experiments 1 and 2, we found that social skill domain of the AQ was negatively correlated with SSI (Figure 5D, Pearson’s *r*(40) = −0.37, *p* = 0.015) and perceptual bias index (Figure 5E, Pearson’s *r*(40) = −0.33, *p* = 0.030), that is, a lower AQ score (higher social proficiency) was associated with a stronger social susceptibility and more selective sensory evidence accumulation toward others’ opinions. A follow-up mediation analysis revealed that individual autistic tendency indirectly affected social susceptibility via the perceptual bias index (a×b = −0.25, SE = 0.11, *p* = 0.039, 95% CI = [-0.45, −0.03]). Moreover, the influence of autistic tendency on social susceptibility was fully mediated by its perceptual processing (Figure 5F, from c = −0.37, *p* = 0.015 to c’ = −0.12, *p* = 0.242). This relationship between personality traits and individual social susceptibility was also confirmed when taking the Big Five personality test^33^(Figure S7). The dimension of extraversion was found to be significantly correlated with perceptual bias index (Pearson’s *r*(40) = 0.34, *p* = 0.029) and marginally significantly correlated with SSI (Pearson’s *r*(40) = 0.27, *p* = 0.081). Taken together, these results suggest that individuals with higher social proficiency (less autistic-like traits) are more susceptible to social influence by promoting a selective sensory evidence accumulation process toward the choices favored by others.

## Discussion

Initiated by Sherif^34^ and Asch’s studies,^13^ a key question in social psychology is whether social influence can alter basic perceptual processes. In this study, we designed a dyadic training paradigm and demonstrated that observing others’ choices can lead to a persistent perceptual change (i.e., shift in visual categorization boundary). We further sought to identify the critical factors that modulate perceptual decisions under such interactive social contexts. We showed that confidence has a great impact on social influence. Using a diffusion model approach, we characterized how individuals integrate social information with confidence into the process of sensory evidence accumulation. In addition, we quantified the individual differences in susceptibility to social influence and linked it to personality traits (e.g., autistic-like tendency). This enables us to provide the following main advances in understanding the computational cognitive mechanisms underlying social influence on perception.

### Long-term effects of social influence on perceptual decision-making

First, our study reveals a robust and long-lasting impact of social influence on a basic perceptual task, complementary to the existing investigation on the long-term effects of social influence focusing on higher-level cognitive processes such as preferences and attitudes.^3^ For instance, it has been reported that social conformity in facial attractiveness judgments lasts for up to 3 days, but not for longer than 7 days. Here, we showed that participants’ perceptual boundaries are significantly shifted after a short-term dyadic training, highlighting individuals’ sensitivity to the opinions of others.^6^^,^^7^^,^^35^^,^^36^ Notably, the observed perceptual changes are still evident in the absence of social context (i.e., test phases) and persist for an extended period of up to six weeks, implying that social influence can be internalized through genuinely altering perception more than merely a temporary compliance with others’ opinions. Interestingly, such social influences are observed even in individuals who have been well-trained for multiple days with trial-by-trial error feedback. Mere 90-min social learning with biased feedback significantly alters their original perceptual boundaries, indicating a robust effect of social influence in shaping our perceptual experience which also occurs in familiar stimuli. However, there was no significant improvement in perceptual sensitivity observed in our study, which suggests that short-term dyadic training without error feedback may not be sufficient to facilitate perceptual sensitivity, given that the amount of training, sleep-related memory consolidation and informative feedback are known to play significant roles in perceptual learning.^37^^,^^38^^,^^39^^,^^40^

Furthermore, our modeling results confirm that during social learning participants boost the accumulation of sensory evidence toward partners’ choices, rather than merely shifting response criteria. In line with previous studies,^6^^,^^7^^,^^14^ our results confirm a perceptual bias caused by social influence. Neuroimaging studies have provided evidence that social influence modulates early sensory processing, such as the visual P1 and N1 components.^41^^,^^42^ Kelly and O’Connell^43^ have found that the buildup of sensory evidence leads to an increase in the lateralized readiness potential (LRP), which is associated with a faster drift rate favoring the majority response.^7^ Edelson et al.^44^ have shown that social influence could even extend to long-term memory by modifying the neural representation of memory. Taken together, short-term dyadic training exerts a robust and enduring influence on perceptual decision-making by internalizing social influence, with sensory evidence accumulation processes genuinely altered.

**Figure 6:**
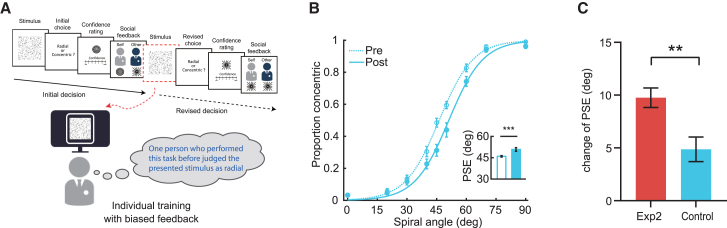
Results of control experiment (A) Experimental design of control experiment. The test and training protocols matched experiment 2, except that the observers were trained individually and provided with the categorical judgments about the same stimulus from an alleged peer advisor. (B) The proportion of responses in which observers indicated the Glass pattern as concentric is plotted as a function of spiral angle before and after training. Data are shown for the pretest (dash lines) and the posttest (solid lines). Insets indicate the corresponding PSEs for each testing phase. (C) Comparison of the shift of categorization boundary (i.e., change of PSE) between the control experiment and experiment 2. Error bars show ±1 SEM.∗∗*p* < 0.01, ∗∗∗*p* < 0.001.

Why do people conform? From a traditional viewpoint, aligning with others’ opinions could be motivated informationally to improve decision accuracy or normatively to affiliate with others for gaining acceptance or maintaining positive self-esteem, referred to as informational and normative influences,^2^^,^^45^ respectively. The current study focuses more on the mechanisms of informational influence, as it was designed to minimize group pressure through the use of a single gender-matched advisor. However, there still exist potential effects of normative influence, as the social context we have introduced in the current design (i.e., interactive training with a partner) may involve a process of social comparison (individuals compare themselves to other people)^40^ or reciprocity (participants reciprocate influence with their partner by gravitating toward the susceptible partner’s opinion)^46^^,^^47^ which is going beyond information aggregation for decision improvement. To provide confirmatory evidence for the contribution of normative influence, we ran an additional control experiment during which the test and training protocols matched experiment 2, except that the observers were trained individually but provided with peer-absent social feedback (Figure 6). This manipulation ensures the informed accuracies well controlled across experiments, and allows us to probe the potential normative influence on perceptual decisions through the environmental settings of online social interaction. Our results show that the biased social feedback, in spite of the absence of a partner, still significantly shifted the observers’ criteria for categorization (PSE at pretest vs. posttest: 45.95 vs. 50.82, 95% CI for the mean difference = [2.44, 7.29], Cohen’s *d* = 0.94, *t* (19) = 4.20, *p* < 0.001). Critically, the observed perceptual change in the control experiment was significantly smaller than that in experiment 2 (ΔPSE: experiment 2 vs. control experiment, 9.75 vs. 4.87, 95% CI for the mean difference = [1.90, 7.87], Cohen’s *d* = 1.03, *t*(39) = 3.31, *p* = 0.002). These results indicate that the observed social conformity is not just a result of informational influence in terms of increasing decision accuracy but also reflects normative influence which might relate to a process of social comparison or reciprocity. In particular, the social context with real-time human interactions exerts a greater social influence.

### Confidence controls social influence integration into perceptual decision-making

Second, our study highlights the critical role of confidence in the integration of social influence into perceptual decision-making. It is worth noting that the observed shift of perceptual boundary after dyadic training with biased social feedback (i.e., change of PSE in experiment 2) was 9.8 ± 0.9° (*M* ± *SE*), which was significantly smaller than an expected PSE shift of 15° induced by classical non-social feedback (i.e., error feedback indicating whether the choices were correct or not) (*t*(20) = −5.67, *p* < 0.001) according to reinforcement learning theory.^48^^,^^49^^,^^50^ Our results suggest that although people are inevitably influenced by the opinions of others, they are not simply following the herd; instead, there exists a processing mechanism which integrates the personal information gathered from sampling the physical inputs, and social information provided by others. To investigate further, our paradigm is unique in measuring self-rated confidence in a two-phase design, allowing us to dynamically monitor how this internal metacognitive process affects subsequent perceptual decision-makings, and interacts with social influence. Our behavioral and modeling results demonstrate that when observers felt less confident in their decisions, they were more likely to conform to the peers’ opinions, and a selective sensory evidence accumulation process in favor of others’ choices was promoted. This cognitive mechanism aligns with recent work, which has demonstrated that high confidence alters the way the brain accumulates new sensory information by overweighting evidence consistent with their beliefs while discounting evidence incompatible with them.^21^^,^^51^ Importantly, tracking individual process of confidence judgments over training (i.e., metacognitive sensitivity) was a robust predictor of perceptual changes (i.e., shift of categorization boundary) at post-training tests and re-tests even six weeks later. These results comply with a “copy when uncertain” social learning strategy which can account for situations wherein individually acquired information is imprecise.^52^^,^^53^ For example, individuals seek less social information and are keen to persuade others when they are confident in their choices.^16^ By contrast, they ask for social advice more often when unsure.^54^ This pattern is similar to a Bayesian interpretation of multisensory integration, which proposes that agents weigh and combine different sensory modalities according to their reliability.^55^ To sum up, we argue that a confidence-based learning principle could be a more generalized rule for information integration, which also applies to social contexts. Specifically, a confidence-regulated sensory evidence accumulation process controls the integration of social influence into perceptual decision-making.

Note that confidence is not an absolute measure. Rather, it may dynamically interact with social contexts. For example, recent work has shown that individuals adjust their confidence relying on others’ choice accuracy.^56^ In collective decision-making, accurate communication of confidence can facilitate performance,^8^ while poor metacognitive ability may lead to biased evidence accumulation and yield worse decisions.^22^^,^^57^^,^^58^ Similarly, we observe a modulation of confidence by social influence in our study. Specifically, conforming to the opinions of others significantly boosts the confidence in decisions. However, it remains debated in this field what information is actually referenced to in metacognition for guiding behavior.^21^ Our results provide some interesting observations regarding this issue. We show that experienced participants are less influenced by others’ opinions. In addition, with informed accuracies well controlled, social contexts involving real-time human interactions exert a greater social influence. These findings suggest that there might exist multi-level processes of metacognition under social influence, more than merely monitoring the accuracy of a decision. Future work might adopt delicate design and modeling approach to disentangle these processes.

### Individual differences in social susceptibility and its correlations with personality traits

Finally, we characterize the individual differences in the extent to which they count on social information when making perceptual decisions. Results show that the individual variation in social susceptibility (i.e., SSI) relates to the bias in sensory evidence accumulation process rather than response preference, confirming again that social influence is associated with the changes in perceptual processing. More importantly, our study provides evidence for the linkage between personality traits and individuals’ susceptibility to social influence. Previous research has shown that children with autism are less likely to conform to misleading advice compared to typically developing children.^24^ Drift-diffusion modeling has suggested that such social influence in neurotypical children is due to changes in sensory processing, but this does not emerge in autistic children.^6^ Here we extend these findings in autistic patients to healthy adults. Our results discover that individuals with higher social proficiency (e.g., lower AQ scores in social skill subscale, higher extraversion of the Big Five personality traits) are more susceptible to social influence by driving a perceptual bias toward social information. It is slightly different from what has been reported in a social conformity study conducted in children,^24^ which underlines the autistic trait of attention to detail as the critical predictor of conformity. One possible explanation for this discrepancy could be result of the developmental differences in the social brain.^59^ The development and maturation of social cognitive abilities are thought of as more sophisticated compared to basic perceptual functions. A recent study has described a developmental trajectory of increasing social influence integration in neurotypical children, with a prominent effect emerging around early adolescence.^6^ It is possible that children focus more on the precise perception of objects while being less sensitive to top-down influence of social information. As the social brain develops, social influence may work through a different mechanism linking more to individual social proficiency in adults.

In sum, combining behavioral methods with computational modeling across a series of parallel experiments, we have developed a comprehensive understanding of how individuals flexibly utilize different information sources when making decisions under interactive social contexts. We have demonstrated that social influence engenders prolonged perceptual changes by altering the process of sensory evidence accumulation, and highlight the critical roles of confidence and personality traits (e.g., autistic traits) in this modulation effect. These findings offer compelling evidence that our perceptual experiences are deeply influenced by social factors, and shed new light on the possible biopsychosocial mechanisms of social influence on perceptual experience.

### Limitations of the study

This work brings to light several topics that warrant further investigation. First, the current study focuses on delineating the level of processing at which personal and social information were incorporated into decision process (e.g., perceptual bias vs. response bias). We lack a detailed understanding of the dynamics of the decision-making process in social contexts. It would be valuable to develop a mechanistic model of social decision-making, accurately capturing how confidence and social information dynamically interact and change during the social learning process. Second, the inferences of cognitive process of social contexts on perceptual decisions are made on a basis of behavioral data, lacking direct biological evidence. Future work may explore the neural substrates that mediate the integration of personal and social information into perceptual decisions. In particular, this social learning mechanism could be associated with an interaction of multiple brain systems involved in perceptual decision-making (e.g., visual areas and lateral intraparietal area), social cognition (e.g., superior temporal sulcus and temporoparietal junction), and metacognition (e.g., dorsal anterior cingulate cortex and prefrontal cortex). It will help uncover the neurocomputational basis underlying how individuals flexibly utilize different information sources to construct adaptable representations of the world and guide adaptive behaviors.

## Resource availability

### Lead contact

Requests for further information and resources should be directed to and will be fulfilled by the lead contact, Rui Wang (wangr@psych.ac.cn).

### Materials availability

This study did not generate new unique reagents.

### Data and code availability

• Pre-processed data for the current study are available at the Psychological Science Data Bank (https://doi.org/10.57760/sciencedb.psych.00170).
• All analytic code has been deposited at the Psychological Science Data Bank (https://doi.org/10.57760/sciencedb.psych.00170).
• Any additional information required to reanalyze the data reported in this paper is available from the lead contact upon request.

## Acknowledgments

We would like to thank Sheng Li for providing the Glass Pattern stimuli and Yupei Liu for help with data validation. This work was supported by grants from the STI2030-Major Projects (2022ZD0208200 and 2021ZD0203800), the 10.13039/501100001809National Natural Science Foundation of China (31701003 and 32430043), the 10.13039/501100004739Youth Innovation Promotion Association of Chinese Academy of Sciences (2018115), the Interdisciplinary Innovation Team (JCTD-2021-06), the 10.13039/501100015956Key Research and Development Program of Guangdong, China (2023B0303010004) and the 10.13039/501100012226Fundamental Research Funds for the Central Universities.

## Author contributions

B.Z.: conceptualization, methodology, software, investigation, formal analysis, visualization, writing – original draft. Y.C.: visualization, writing – original draft. R.W.: conceptualization, methodology, formal analysis, supervision, writing – original draft, writing – review and editing. Y.J.: conceptualization, methodology, supervision, writing – review and editing.

## Declaration of interests

The authors declare no competing interests.

## STAR★Methods

### Key resources table

**Table:** 

REAGENT or RESOURCE	SOURCE	IDENTIFIER
**Deposited data**		

Behavioral data	This paper	https://doi.org/10.57760/sciencedb.psych.00170

**Software and algorithms**		

MATLAB (R2021a)	MATLAB software	http://www.mathworks.com/products/matlab/
Psychtoolbox-3	Brainard et al.^60^	http://psychtoolbox.org/
Python package HDDM (v 0.8.0)	Wiecki et al.^29^	https://hddm.readthedocs.io
SPSS (v 26.0)	IBM SPSS Statistics	https://www.ibm.com/spss
PROCESS (v 3.4.1)	Hayes^61^	http://www.processmacro.org/
Jamovi (v 2.3.21.0 )	Şahin et al.^62^	https://www.jamovi.org/
Analytic code	This paper	https://doi.org/10.57760/sciencedb.psych.00170

#### Experimental model and study participant details

A total of 104 college students took part in this study and they were randomly allocated into different experimental groups. One participant was excluded in Experiment 1 due to technical issues, and another participant in Experiment 2 dropped out due to a scheduling conflict. The final sample consisted of 102 young adults (42 males and 60 females, mean age = 23.0 ± 2.6 years) who completed the experiments. The sample size for each experiment was as follows: Experiment 1: n=21, Experiment 2: n= 21, Experiment 3: n=20, Experiment 4: n=20, and control experiment: n=20. The sample size was determined based on a prior power analysis (matched-pairs t-test, two-tailed) using G∗Power 3.1.^63^ Considering there was no previous study exactly matching the current experimental design, we therefore chose a medium effect (Cohen’s *d* = 0.70) with alpha at 0.05 and power of 80%. The calculation revealed that a sample size of at least 19 observers, we further increased the sample size to ∼20 in each group. All participants were of Asian descent. Ancestry was not collected, and the authors do not expect this detail to have potential impact on the results reported here. All participants were naive to the aim of the study, had a normal or corrected-to-normal vision, and gave written informed consent in accordance with procedures approved by the institutional review board of the Institute of Psychology, Chinese Academy of Sciences (Protocol Number: H17029).

### Method details

#### Stimuli and task

Observers were presented with Glass patterns, generated using previously described methods.^48^^,^^49^ In particular, stimuli consisted of white dot pairs (dipoles) displayed within a square aperture (size = 7.7 ° × 7.7 °) against a black background. The size of each dot was 2.3 × 2.3 arc min^2^ and the dot density was 3% with the Glass shift (i.e., the distance between two dots in a dipole) of 16.2 arc min. To generated patterns, intermediate between radial and concentric, dipole angles were parametrically varied from 0° (radial pattern) to 90° (concentric pattern) (Figure 1A). Each stimulus comprised signal dpoles that were aligned according to the specified spiral angle, and noise dipoles for which the spiral angle was randomly selected. Observers were presented with 60% signal Glass patterns (spiral angles = 0°, 20°, 30°, 40°, 45°, 50°, 60°, 70°, and 90°) and performed a categorization task indicating whether the viewed stimulus was radial or concentric. They were requested to respond as quickly and accurately as possible. The presentation of clockwise and counterclockwise patterns was randomized across participants. A new pattern was generated for each trial, resulting in local jittering in stimulus position. Experiments were controlled using MATLAB (The MathWorks, Natick, MA) and the Psychophysics toolbox 3.^60^ Stimuli were presented on a 23-inch LCD monitor (1920×1080 pixels, 60 Hz frame rate) at a distance of 67 cm.

#### Experiment 1

We recruited observers in pairs (gender-matched), and they arrived at the laboratory simultaneously. The two observers met briefly and had their portrait photos taken by the experimenter. They were arranged to enter two adjacent laboratories. Experiment 1 included three stages: pretest, training, and posttest. We developed a dyadic training paradigm in which pairs of observers were asked to perform the visual categorization task simultaneously and each one was informed of his partner’s responses during training. Unbeknownst to them, the peers’ responses were simulated using a computer algorithm. Pretest and posttest took place immediately before and after training. All participants were tested singly during test stages. Before the formal experiment, observers were familiarized with the stimuli and task in a short practice session during which they were shown 100% signal Glass patterns and were asked to categorize the stimuli as either radial or concentric patterns.

During the training stage, each trial consisted of two phases: an initial decision phase and a revised decision phase (Figure 1B). A red central fixation lasting 1000 ms indicated the start of a trial. The initial decision phase started with a 500 ms black fixation, after which a 300 ms Glass pattern stimulus (60% signal) was presented. Observers were asked to make a two-alternative forced choice (2AFC) to judge whether the stimulus was radial or concentric. They were then required to report their confidence in this choice on a numerical scale from 1 (low confidence) to 7 (high confidence). Afterwards, observers were informed of their partner’s choice about the same stimulus; both observers’ and the alleged partners’ choices were displayed underneath their corresponding portraits for 1500 ms. It was worth mentioning that the partners’ choices were actually generated by a psychometric function (Slope = 0.2, PSE = 45 deg). In the subsequent revision phase, the procedure followed the same timeline as the initial decision phase. Observers had the opportunity to revise their decision and confidence rating after viewing a second Glass pattern stimulus with the same spiral angle as the stimulus in the initial decision phase. Note that prior to training, the observers had been informed that the stimuli had exactly the same spiral angle (though different appearance) at both decision phases for the same trial. The training stage lasted approximately 1.5 hours and consisted of 5 blocks. Each block comprised 90 trials, with 10 trials for each spiral angle.

Before and after the training stage, we tested observers’ ability to categorize global form patterns. For each trial, observers were presented with a Glass pattern stimulus (60% signal) for 300 ms and then made a categorical judgment (radial or concentric). Each test consisted of 180 trials, with 20 trials for each of the nine spiral angles. No any feedback was provided during test stages.

#### Experiment 2

Experiment 2 followed the same design and procedure as in Experiment 1, except that the PSE parameter of the psychometric function which simulated the partner’s choices was adjusted to 60°, referring to a biased category boundary. To examine whether the observed changes in categorical boundary after short-term dyadic training was maintained over time, seventeen observers of Experiment 2 were called back for an additional re-test (180 trials) six weeks after dyadic training (spaced by 45.4 ± 8.5 days on average).

#### Experiment 3

Experiment 3 tested whether observers' perceptual sensitivity and categorization boundary were altered during individual training. During the training stage, observers performed the categorization task similar to Experiment 1, except that there was no any feedback provided. That is, for each trial, observers categorized the global form pattern of the same spiral angle twice (initial and revised decisions) but did not receive any social feedback. The test stages were identical to Experiment 1.

#### Experiment 4

Experiment 4 explored whether experienced observers with less ambiguous categorization boundaries are still influenced by biased social feedback. Observers underwent five stages over multiple days, that is a pre-training test, individual training, an intermediate test, dyadic training, and a post-training test. After a brief pre-training test, observers were trained individually for a minimum of two and a maximum of four sessions conducted on consecutive days (1800-3600 trials in total) to improve their perceptual sensitivity in this visual categorization task. For each trial, the stimulus was presented for 300 ms and observers were asked to decide whether the viewed stimulus was radial or concentric. If they made an incorrect choice, audio error feedback was provided. Each training session comprised five blocks with 180 trials per block. The last three stages were conducted on the last day and the procedure matched to Experiment 2. Following a similar training protocol, observers were trained in pairs with biased social feedback using an algorithm based on a shifted categorization boundary. For a better control, the simulated biased categorization boundary was set in opposite directions, either 60° or 30° in this experiment. Observers were randomly assigned to the 60° boundary group or to the 30° boundary group.

#### Control experiment

To examine whether the observed behavioral conformity to the opinions of others in this social context (interactive training with a partner) was just a result of informational influence in terms of increasing decision accuracy, we ran an additional control experiment (Figure 6). The procedure including the presentation of stimuli and feedback matched Experiment 2, except that the observers were trained individually but provided with peer-absent social feedback. Observers were told that during training they would see the opinions of a gender-matched peer advisor about the same stimulus, and this advisor was randomly selected from the participants who had performed this task before. In reality, each observer was coupled with a computer algorithm just like Experiment 2. This manipulation ensured the informed accuracies well controlled across experiments, and allowed us to probe the normative influence under the social context of real-time interacting with human on perceptual decision-making going beyond information aggregation for decision improvement.

#### Questionnaire

##### Quantifying individual traits

Before the formal experiment, individual traits were assessed for each observer using the adapted version of the Autism-Spectrum Quotient (AQ)^32^ and the Chinese Big Five Personality Inventory brief version (CBF-PI-B).^33^

##### Debriefing

After finishing the posttest following dyadic training, observers in Experiments 1, 2, and 4 were required to complete a debriefing form that assessed the appraisals of their own and partner’s performance. Most observers reported that their performance was comparable to that of their partners. All observers stated that they believed they were working with another human observer in the neighboring room.

### Quantification and statistical analysis

#### Psychometric functions

We calculated the proportion of concentric responses to stimulus spiral angle and fitted them with a Boltzmann sigmoid function: f(x)=1/(1+exp(−a×(x−c))). The spiral angle corresponding to a probability of 50% point on the psychometric function is the perceptual boundary (*c*) or the point of subjective equality (PSE). A PSE of 45° means consistency between the perceptual and physical boundary of the stimulus. The slope (*a*) of the fitted sigmoid function is an index of one’s perceptual sensitivity in the global form patterns discrimination task.

#### Hierarchical drift-diffusion model

Drift diffusion model fitting was implemented in Python 3 using the hierarchical drift-diffusion model (HDDM) toolbox.^29^ In the hierarchical DDM, subject parameters were drawn from a group-level distribution, and the posterior distribution of each parameter at both subject and group levels was simultaneously estimated using Markov-Chain Monte-Carlo methods. The model fitting was performed using stimulus coding such that decision boundaries corresponded to those for radial and concentric pattern categorizations. In the DDM framework, choice bias can be modeled in two ways: first, the starting point can be biased towards the preferred choice, representing a response bias; alternatively, the drift rate can be altered to induce a bias in the evidence accumulation processing, showing selective sensory information uptake (i.e., perceptual bias). Therefore, we assumed that social information (partner's choices; concentric pattern, -1; radial pattern, 1), and personal information (initial confidence signed with their categorization; concentric, negative; radial, positive; parametrically ranging from -1 to 1) would modulate the starting point and drift rate parameters. As stimulus uncertainty particularly affects the drift rate parameter,^19^ we also included dependency of the drift rate on stimulus uncertainty (0 to 90°, parametrically ranging from 1 to -1). Finally, to investigate how personal or/and social information may modulate the starting point and drift-rate parameters, we used regression analysis and compared 10 DDMs with different parameter constraints (see below). Drift rate varied with stimulus uncertainty in all model.

Model 1: No effect of social and personal information allowed on the starting point and drift rate parameters (baseline model).

Model 2: Starting point, *z*, only depended on personal information.

Model 3: Drift rate, *v*, only depended on personal information.

Model 4: Starting point and drift rate depended on personal information.

Model 5: Starting point, *z*, only depended on social information.

Model 6: Drift rate, *v*, only depended on social information.

Model 7: Starting point and drift rate depended on social information.

Model 8: Starting point, *z*, depended on both personal and social information.

Model 9: Drift rate, *v*, depended on both personal and social information.

Model 10: Starting point and drift rate depended on both personal and social information (full model).

As we focused on how confidence and social information modulate subsequent perceptual decisions, the DDM analysis was only applied to the revised decisions during dyadic training in Experiments 1 and 2 following a similar approach as in recent work.^22^ Observers’ confidence ratings on their initial decisions, which closely track the precision and reliability of sensory evidence accumulation,^64^^,^^65^ are considered representative of observers’ subjective independent judgments (i.e. personal information) before receiving any social feedback. All trials with RTs greater than 3.5 s were excluded before model fitting (across all experiments, 152 trials (0.54%) were excluded, with a mean response duration of 6.4 s) and the outlier probability was set to 5%.^29^ Both group and subject-level parameters (starting point, drift rate, decision threshold, and non-decision time) were estimated in model fitting. Model fits were assessed by comparing Deviance Information Criterion (DIC) values which had a degree penalty for model complexity. All DDMs were estimated with MCMC method (50,000 samples; burn-in = 10,000; thinning = 10).

#### Model analysis

To better understand the cognitive mechanisms how personal and social information modulates perceptual decision-making, we tested a series of candidate models within the DDM framework and demonstrated that the best-fitting model is the full model which incorporates dependencies of the starting point and the drift rate on both personal and social information. We took several steps to validate this best-fitting model (see details below). First, this full model provided a good fit to the behavioral data (i.e., RT distributions and perceptual choices) at both group and individual levels (Figures S2–S4). We also tested the validity of this model with a parameter recovery analysis to ensure that the key model parameters described individual differences and were interpretable, rather than an artifact of the parameter optimization procedure (Figure S5). In addition, a cross-validation analysis was performed, wherein datasets with biased and unbiased social feedback were generated using parameters from Experiment 1 (trained with unbiased feedback) and Experiment 2 (trained with biased feedback), respectively. These simulated results successfully reproduced the behavioral patterns of perceived categorization boundaries varied with the partners’ perceptual boundaries (Figure S6).

##### Posterior predictive checks

Because a superior relative model fit does not necessarily mean that the winning model captures key aspects of the real data, we additionally performed posterior predictive checks. To this end, we generated 2000 full datasets from the best-fitting model, based on the posterior distribution of the parameters. To compare these simulated data to the observed data, we averaged over all simulations to obtain the average reaction time and probability of switching in the revised decision, separately for the different trial types (high confidence and agreement, low confidence and agreement, high confidence and disagreement, low confidence, and disagreement; Figures S2C–S2F). Furthermore, to visualize how models accounted for the overall observed RT distributions, the simulated datasets were smoothed via non-parametric density estimation and overlaid on the observed RT distributions for each trial type (Figures S2D and S2F). To visualize how the model fits deviated from the data when the starting point or drift rate term was independent of personal and social information, we also simulated model predictions from the fits of other model families (Figure S3). We also assessed how well the model accounts for the RT distributions at the subject level. For each observer, we overlay the distribution of simulated reaction times with the true reaction time distributions, separately for radial and concentric responses (Figure S4).

##### Parameter recovery analyses

To verify that the effect of personal and social information on drift rate and starting point reflected true individual differences in perceptual decisions, as opposed to an artifact of the parameter optimization procedure, we checked the capacity of recovering the correct parameters using simulated datasets. We generated ten datasets, generating parameters based on the subject-level posterior distributions, and re-fit these datasets with the generating model using the same methods as outlined above. The recovery of subject-level parameters was examined by plotting the correlations between generating and estimated parameters (Figure S5).

##### Model simulations

To validate that our winning model (model 10) could effectively capture behavioral patterns of Experiments 1 and 2, we used model simulation to cross-validate these inconsistent results from these two experiments. The differences in behavioral results between Experiment 1 and Experiment 2 were mainly due to differences in the PSE of the simulated partners. Therefore, we generated 2000 full datasets, each dataset was generated with parameter values sampled from the posterior distribution, but changed the partners’ perceptual boundary (45° or 60° spiral angle) to predict how individuals’ PSEs vary with partners’ perceptual boundary. To compare the simulations to real data in Experiments 1 and 2, we averaged over the simulations to obtain concentric responses at each spiral angle and plotted psychometric curves (Figure S6).

To examine whether individuals’ decision criteria would vary with their confidence and sensitivity to social information when interacting with a partner with categorization bias (60° spiral angle). We created two 2-dimensional parameter grids, each for starting point and drift rate. Each parameter grid contains the average effect of personal (range from 0.5 to 2, in increments of 0.15) and social information (range from 0 to 0.8, in increments of 0.08) on these parameters. The ranges of these parameters were derived from the posterior distribution of the winning model. We generated 10,000 trials at each spiral angle at each combination of parameters. The simulated responses were averaged to obtain the proportion of concentric responses at each spiral angle, and the change of PSE was calculated for each parameter combination.

#### Regression analysis

##### Metacognitive sensitivity

We quantified the metacognitive sensitivity in each observer using trial-by-trial linear regression following previous methods.^31^ Specifically, we used accuracy (correct responses, 0; incorrect responses, 1; all responses at the physical boundary (45°) were encoded as 0.5) to predict confidence scores, in which the coefficient of accuracy, that is, metacognitive sensitivity represented the extent to which confidence discriminates between correct and incorrect trials.

##### Social susceptibility index

We quantified the contributions of social information and personal information to revised confidence using trial-by-trial multiple linear regression, in which we predicted the z-scored observer’s signed revised confidence (concentric, negative; radial, positive) using the z-scored observer’s signed initial confidence and the partner's choice (radial, 0.5; concentric, -0.5). Further, we calculated the difference between these two coefficients (β_social_ – β_personal_) to measure a social susceptibility index (SSI). The SSI represents the relative extent of the impact of social information on revised confidence compared to personal information. We extracted the individual parameters estimated from the DDM model and analogously calculated the difference between *v*_social_ and *v*_personal_ or *z*_social_ and *z*_personal_, indicating the perceptual or response bias index in perceptual decision-making.

#### Mediation analysis

We conducted a mediation analysis using the PROCESS^61^ in SPSS version 26 (IBM Corp., Armonk, NY), with individual autistic tendency as the independent variable, perceptual bias index (*v*_social_ - *v*_personal_) as the mediator, and the SSI as the dependent variable. We performed a bias-corrected bootstrap estimation analysis with 5000 samples to calculate the mediation effect. The analysis yielded 95% confidence intervals for the indirect effects (a×b). Statistical significance was set at *p* < 0.05 when the 95% CIs did not include zero.
