# Supplementary material for: Prolonged visual perceptual changes induced by short-term dyadic training: The roles of confidence and autistic traits in social learning
Source: iScience. 2024 Dec 30;28(2):111716. doi: 10.1016/j.isci.2024.111716 (PMC11783384; doi:10.1016/j.isci.2024.111716)
Supplement: Document S1. Figures S1–S7 [file mmc1.pdf]

iScience, Volume *28*

Supplemental information

Prolonged visual perceptual changes induced by short-term dyadic training: The roles of confidence and autistic traits in social learning

Bin Zhan (占斌), Yujie Chen (陈玉洁), Rui Wang (王蕊), and Yi Jiang (蒋毅)

Supplemental information

Supplemental figures

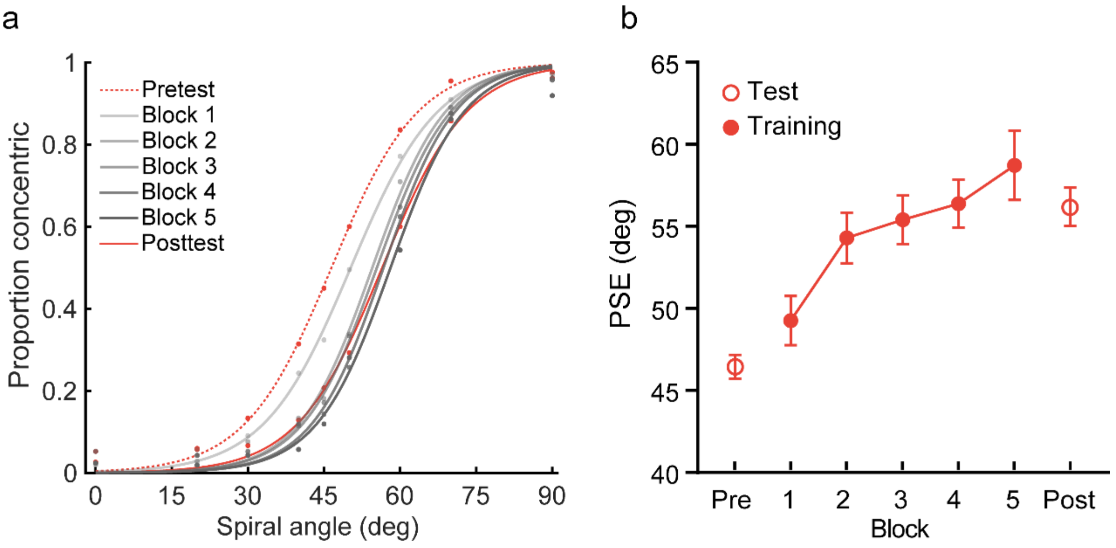

Figure S1. Visual categorization performance across training and test blocks in Experiment 2. (**a**) The proportion of responses in which observers indicated the Glass pattern as concentric is plotted as a function of spiral angle at training (grey lines) and test (red lines) stages. (**b**) PSEs are shown for training (solid circles) and test (open circles) blocks. Training data include human responses collected in the initial decision phase, reflecting observers' subjective independent judgments before exposure to others' choices.

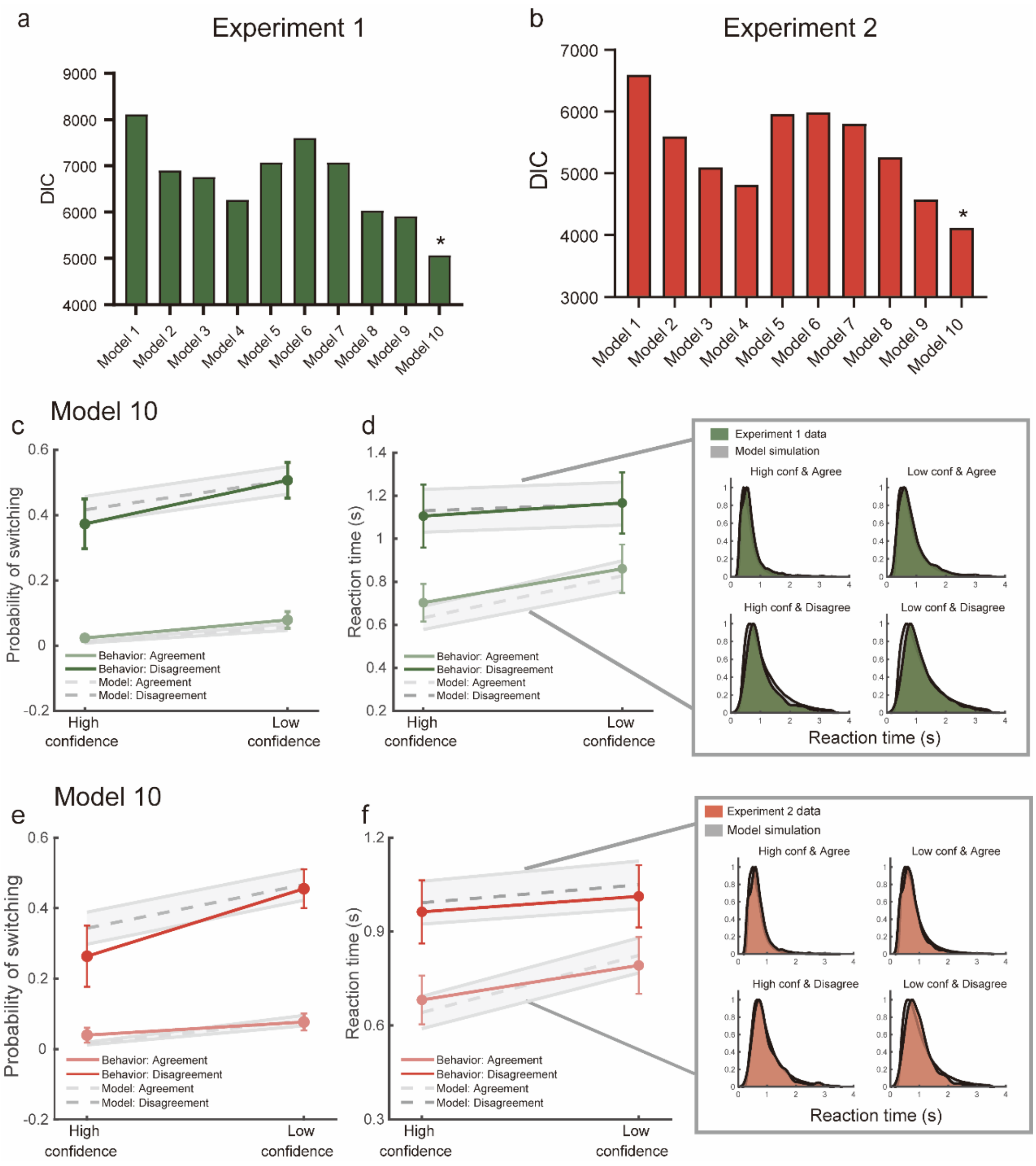

Figure S2. Model comparison and model simulations using the best-fitting model. Model comparison of the 10-alternative drift-diffusion models in Experiment 1 **(a)** and Experiment 2 **(b)**. Models were compared based on the DIC where lower values indicate a better fit. Model 1 is a baseline model with no dependencies on drift-rate or starting point. Models 2-4 represent a model family in which the drift-rate and/or starting point are affected by personal information (initial confidence). Models 5-7 represent a model family in which the drift-rate and/or starting point are affected by the social information. Models 8-10 represent a model family in which the drift-rate and/or starting point are affected by personal and social information. Model simulations (of

the best fitting model) reproduce behavioral patterns of changes of mind **(c, e)** and reaction times **(d, f)** of the second decision in Experiments 1 and 2 when plotted as a function of the social influence and initial confidence. Model simulations are shown as dotted lines and behavioral data as solid lines. Data are presented as mean values ±95% confidence intervals. The righthand panel of **(d, f)** plots the full distribution of reaction times and model predictions for the different trial types (high confidence and agreement, low confidence and agreement, high confidence and disagreement low confidence and disagreement).

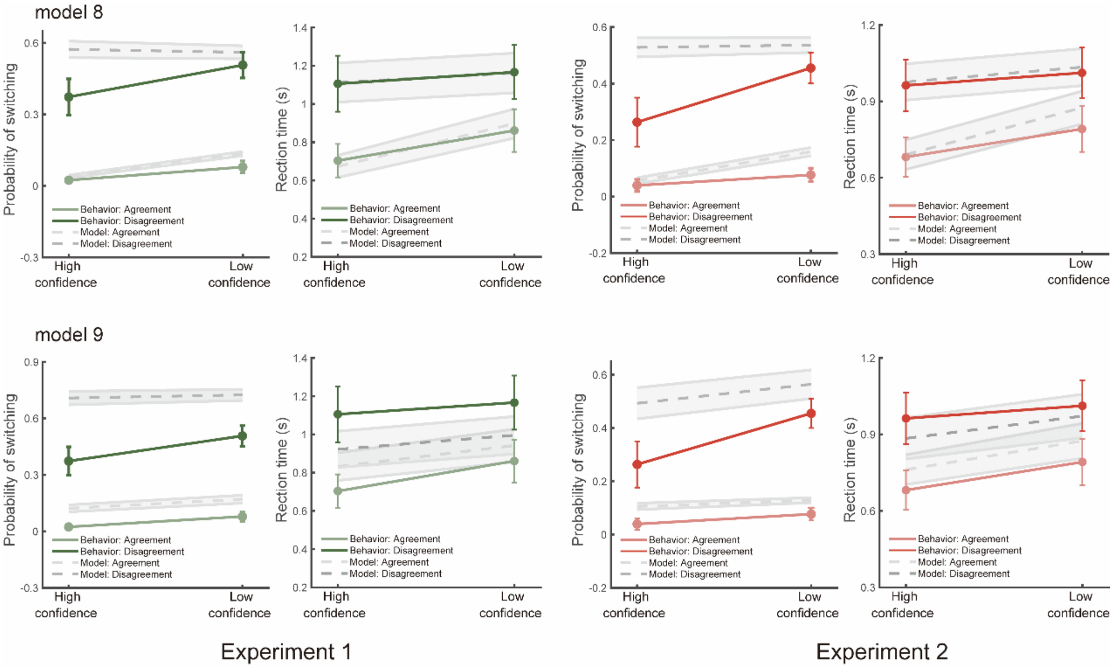

Figure S3. Simulations of alternative drift-diffusion models. To visualize how the model fits deviated from the real data when the dependencies of the starting point or drift rate term were omitted, we also simulated model predictions from the fits of other model families (models 8 & 9). Simulations of alternative models in Experiment 1 (lefthand panel) and 2 (righthand panel). Model simulations are shown as dotted lines and behavioral data as solid lines. Data are presented as mean values ±95% confidence intervals.

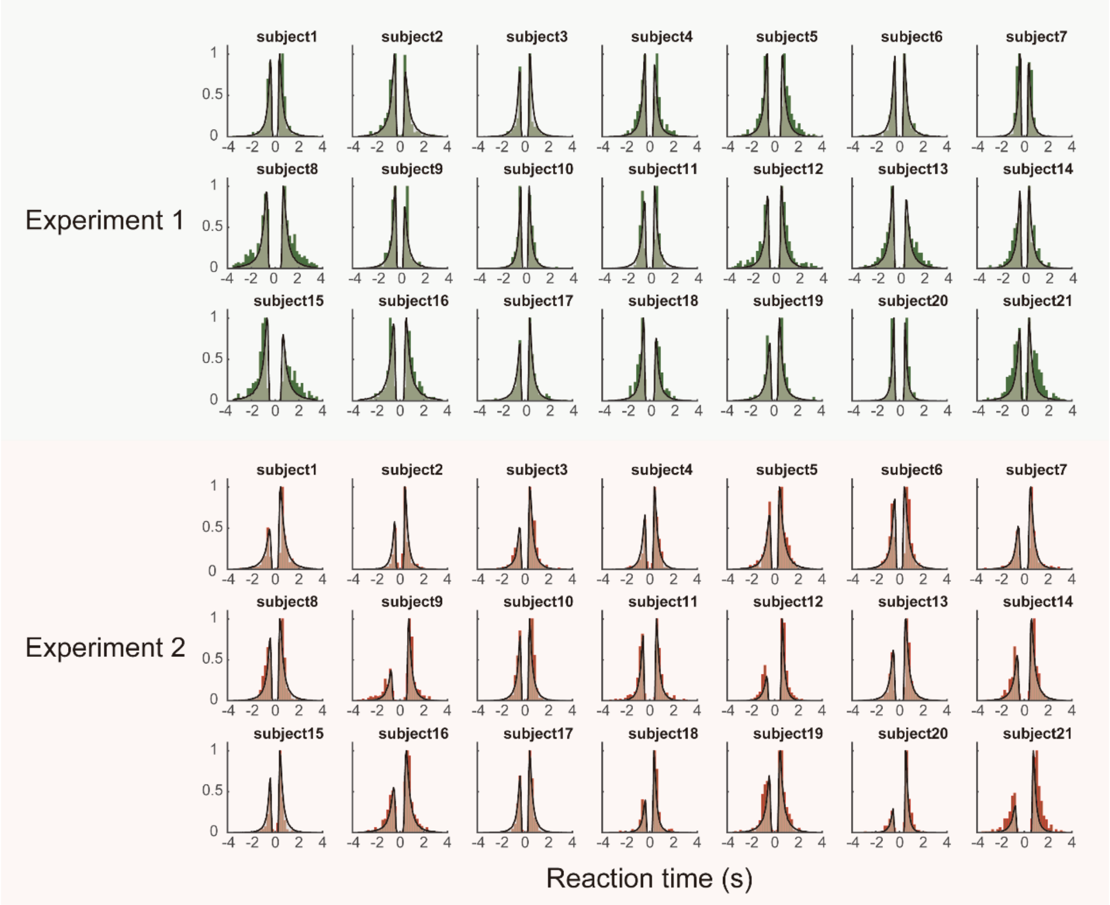

Figure S4. Observed and predicted choice and RT distributions for each participant. For each participant, we plot the RT distributions for concentric (negative RTs) and radial responses (positive RTs) in Experiment 1 (upper panel) and 2 (lower panel). The black line indicates model-predicted choice and RT distributions.

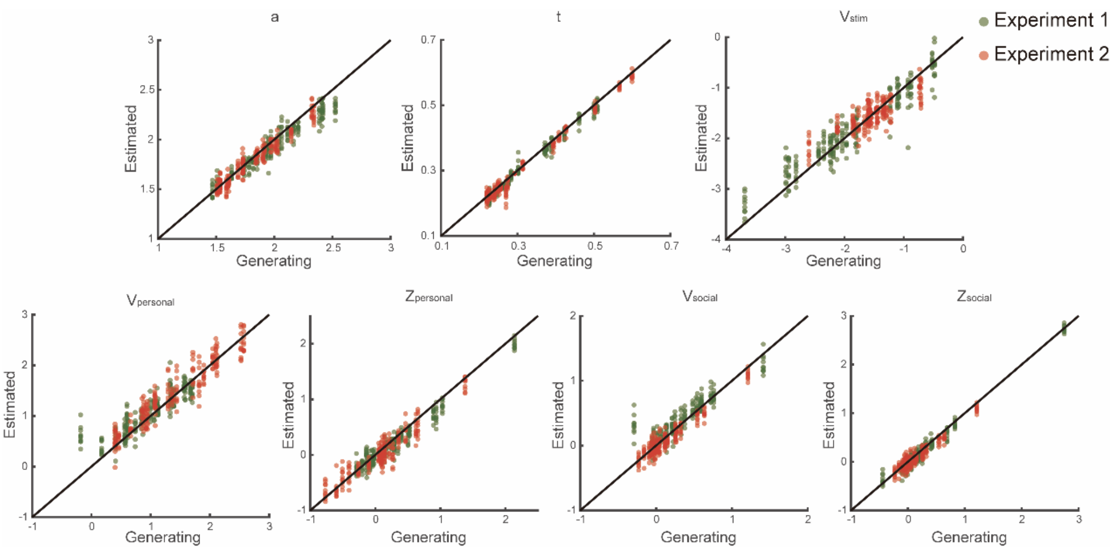

Figure S5. Parameter recovery results for the best-fitting model. Recovery of subject-level model parameters pooled across all ten simulations. Correlation between the generating (true) parameter values (x-axis) and the estimated values (y-axis). A decision threshold, t non-decision time, V_{stim} Drift-rate ~ stimuli uncertainty, $V_{personal}$ Drift-rate ~ personal information, V_{social} Drift-rate ~ social information, $Z_{personal}$ Starting-point ~ personal information, Z_{social} Starting-point ~ personal information.

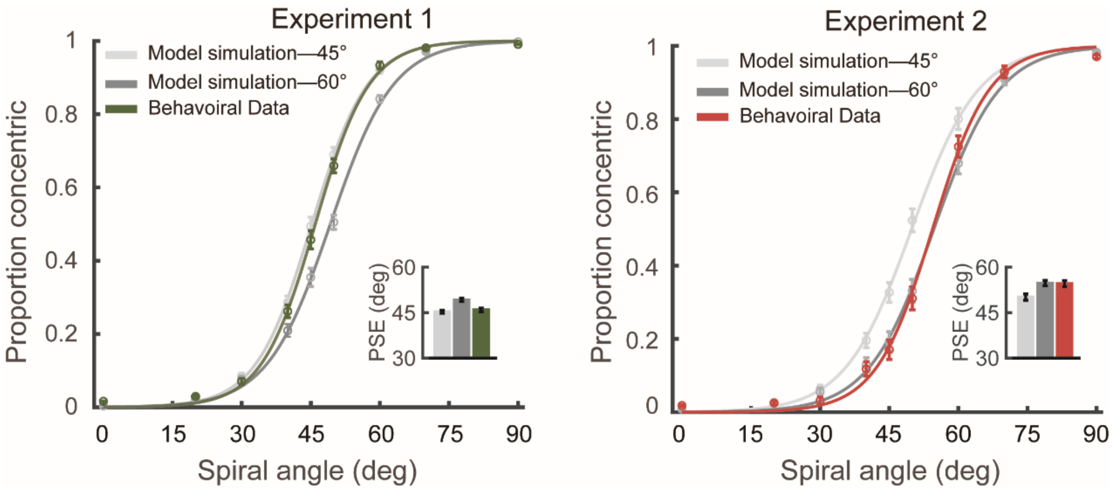

Figure S6. Cross-validation results for the best-fitting model. The behavioral data (generated from the actual experiments) fitted by the psychometric function were plotted with green and red lines. The data predicted by model simulation were plotted with light gray (simulated unbiased social feedback, 45°) and dark grey (simulated biased social feedback, 60°) lines. Insets indicate the corresponding PSEs. Error bars show standard errors of the mean.

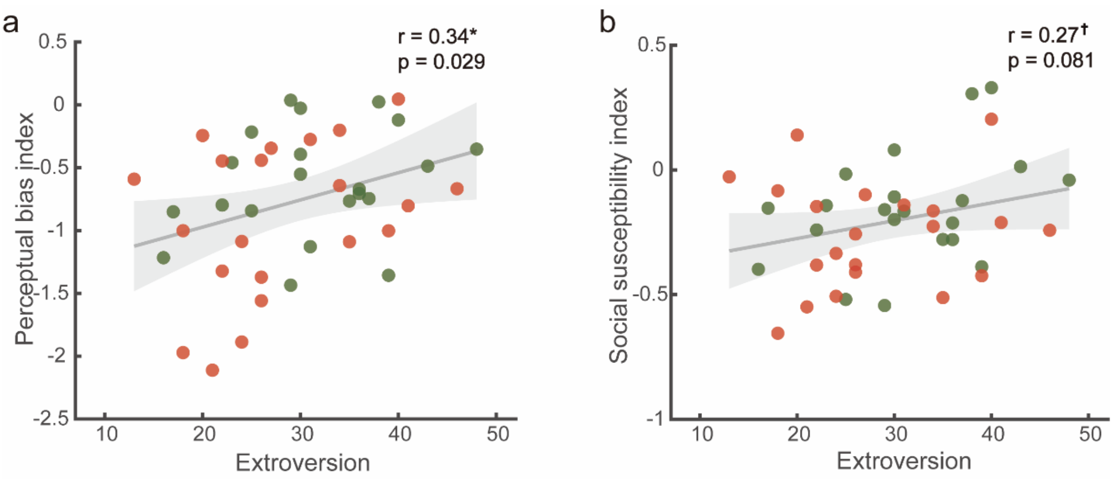

Figure S7. Correlating extroversion with perceptual bias index and SSI. (a) Correlation between Extroversion domain of the Big Five Personality and perceptual bias index (v_{social} - $v_{personal}$). **(b)** Correlation between Extroversion and SSI. Pearson correlation (two-tailed); SSI, social susceptibility index. *$p < 0.05$, † $p < 0.1$.
